# Potential Cancer Risk in Patients with Rheumatoid Arthritis: A Longitudinal Korean Population-Based Analysis

**DOI:** 10.3390/jpm12060965

**Published:** 2022-06-13

**Authors:** Hyo Geun Choi, Ho Suk Kang, Hyun Lim, Joo-Hee Kim, Ji Hee Kim, Seong-Jin Cho, Eun Sook Nam, Kyueng-Whan Min, Ha Young Park, Nan Young Kim, Mi Jung Kwon

**Affiliations:** 1 Department of Otorhinolaryngology-Head & Neck Surgery, Hallym University Sacred Heart Hospital, Hallym University College of Medicine, Anyang 14068, Korea; pupen@naver.com; 2 Department of Internal Medicine, Division of Gastroenterology, Hallym University Sacred Heart Hospital, Hallym University College of Medicine, Anyang 14068, Korea; hskang76@hallym.or.kr (H.S.K.); hlim77@hallym.or.kr (H.L.); 3 Department of Medicine, Division of Pulmonary, Allergy and Critical Care Medicine, Hallym University Sacred Heart Hospital, Hallym University College of Medicine, Anyang 14068, Korea; luxjhee@gmail.com; 4 Department of Neurosurgery, Hallym University Sacred Heart Hospital, Hallym University College of Medicine, Anyang 14068, Korea; kimjihee.ns@gmail.com; 5 Department of Pathology, Kangdong Sacred Heart Hospital, Hallym University College of Medicine, Seoul 05355, Korea; apilas@hanmail.net (S.-J.C.); esnam@kdh.or.kr (E.S.N.); 6 Department of Pathology, Hanyang University Guri Hospital, Hanyang University College of Medicine, Guri 11923, Korea; kyueng@gmail.com; 7 Department of Pathology, Busan Paik Hospital, Inje University College of Medicine, Busan 47392, Korea; hy08.park@gmail.com; 8 Hallym Institute of Translational Genomics and Bioinformatics, Hallym University Medical Center, Anyang 14068, Korea; honeyny78@gmail.com; 9 Department of Pathology, Hallym University Sacred Heart Hospital, Hallym University College of Medicine, Anyang 14068, Korea

**Keywords:** rheumatoid arthritis, malignancy, cancer, hazard ratio, incidence, nationwide longitudinal follow-up study

## Abstract

The potential link between rheumatoid arthritis (RA) and cancer incidence needs to be validated due to inconsistent results between Asian and Western countries. We explored the long-term association of RA with the overall and organ-specific cancer incidence using nationwide population data. This longitudinal follow-up study (2002–2015) included 3070 patients with RA and 12,280 controls (1:4 propensity score-matched for sex, age, residence, and income) from the Korean National Health Insurance Service-Health Screening Cohort database. A Cox proportional hazard model estimated the hazard ratio for malignancy following adjusting for covariates. Despite the similar overall cancer incidence between RA and control groups, differences in the incidence of organ-specific cancers were noted: the RA group had a 1.63-fold greater likelihood for lung cancer (95% confidence interval 1.11–2.40). In the sex-stratified subgroup analyses, the male RA patients exhibited higher odds of lung and thyroid cancer but a lower probability for colorectal cancer; no such associations were detected in either female patients with RA or age subgroups. In summary, the higher likelihood for lung cancer in Korean RA patients, especially thyroid and lung cancer in male RA patients, seems to be characteristic, which needs to be carefully monitored.

## 1. Introduction

Rheumatoid arthritis (RA) represents an immune-mediated, chronic, systemic inflammatory disorder that causes functional disability and early death [1]. Although RA can present at any age, an increased risk with age, especially in individuals older than 50 years, and a 3.5-fold female preponderance has been reported [2]. Moreover, RA characteristically presents in the context of several comorbidities, including malignancy. The substantial increase in the risk for malignancy, especially within the first decade after an RA diagnosis [3,4], and the potential cancer-related mortality risk with age is a concern. Malignancy is the highest reason for deaths in patients suffering RA in East Asian countries, including South Korea, Japan, and the People’s Republic of China, which is remarkably different from Western countries where cardiovascular disease is the leading cause of mortality [5]. Knowledge of the overall and organ-specific RA-associated malignancy risk is of importance to develop and implement preventive strategies for individuals at risk.

In South Korea, given the likely increase in the risk of RA due to population aging and the resultant increase in the incidence of RA-associated cancer, RA might become a public health concern [1]. Previous meta-analyses presented a high risk for lymphoma and lung cancer and a low risk for breast and colorectal cancers in patients with RA [6,7,8]. However, the RA-associated cancer risk varies much more according to age, sex, ethnicity, geographic region, environmental factors, and genetic predisposition [3,4,8,9,10], which the abovementioned meta-analyses did not capture in their analyses [6,7]. Breast and lung cancers were revealed as the most frequent ones in a China-based registry of RA [11], whereas a Taiwanese population-based study reported hematologic malignancies as the highest [3]. Melanoma and non-melanoma skin cancer were identified as the highest ones using US-based data [8], whilst the RA patients in California tended to have the highest risk for hematologic malignancy [9]. Far from those incidences, the most common cancers in Korea include thyroid and lung cancers [12]. In Korea, only three epidemiologic studies have identified the incident malignancy odds of patients with RA; however, it is controversial whether RA is connected with elevated hazards of specific types of cancer, with some studies detecting an increased risk whereas others do not [12,13,14]. Due to the deficient non-RA counterpart participants and inconsistent risk magnitudes of malignancy in the abovementioned studies, the incidence and risk for overall or specific malignancy remains unclear in Korean patients with RA; this aspect should be clarified from long-term follow-up data. Furthermore, as the incidence of both malignancy and RA increases with age, both of these conditions may share certain risk factors or reciprocal associations, and the RA-associated risk of incident malignancy should be verified in a long-term follow-up study after adjusting for potential shared confounders.

We formulated a hypothesis that RA, depending on age or sex, influences the overall incidence of organ-specific cancer likelihood. In this study, we explore the possible correlation of RA with incident malignancy to guide an appropriate cancer-preventive therapeutic strategy based on the cancer risk of newly diagnosed patients suffering RA in the era of personalized medicine.

## 2. Materials and Methods

### 2.1. Ethical Approval

The Institutional Review Board in Hallym University (IRB No. 2019-10-023) approved this study, and the requirement for written informed consent has been exempted.

### 2.2. Study Design

The current longitudinal follow-up study used information from the nationwide Korean National Health Insurance Service-Health Screening Cohort (KNHIS-HSC) database, which offers population-based data to researchers for use in policy or academic studies. The KNHIS is a mandatory nationwide health insurance policy in South Korea that has embraced the medical expenses of more than 98% of all Korean citizens since 1999. Data provided from the KNHIS-HSC database were de-identified to ensure patient anonymity. The information in the KNHIS-HSC database is based on the International Classification of Diseases, 10th Revision, Clinical Modification (ICD-10-CM) diagnostic codes, and the KNHIS-HSC database has been described previously [15].

### 2.3. Definition of Rheumatoid Arthritis

Using the equivalent ICD-10 diagnostic and prescription codes, the data of all patients diagnosed with RA were pulled from the medical claims database. RA was defined as the presence of an RA diagnosis (M05 [seropositive rheumatoid arthritis] or M06 [other rheumatoid arthritis]) on more than two entries in the database, as well as the prescription of biologics or any disease-modifying anti-rheumatic drugs (DMARD) [16,17]. The medications used in the patients included leflunomide, adalimumab, certolizumab, etanercept, golimumab, infliximab, and rituximab. 

### 2.4. Definition of Cancers

In this study, the incidence of the following 10 cancer types was investigated: gastric cancer (ICD-10 codes (C16.0–C16.9), thyroid cancer (C73), colorectal cancer (C18–C21 and D010–D013), lung cancer (C34 and D022), hepatic cancer (C22 and D015), bladder cancer (C67 and D090), pancreatic cancer (C25 and D017), gallbladder and biliary duct cancer (C23 and C24), kidney cancer (C64), and hematologic malignancy (C81–C96). Participants who were repeatedly assigned the same ICD-10 codes more than twice from more than two clinic visits were identified as patients with cancer. 

### 2.5. Participant Selection 

The patients with RA between 2002 and 2015 were sampled from the KNHIS-HSC database. The index date of each patient with RA was designated as the very day when the RA diagnostic ICD-10 codes were electronically allocated to the patients in the health insurance claim database. Any individuals who were diagnosed with cancer before RA were ruled out (*n* = 79). We eliminated patients with RA who were diagnosed within 2002 (1-year washout period, *n* = 1079), as we possibly might include pre-existing RA prior to the index date in the analysis.

The comparison group of people without a diagnosis of RA from 2002 to 2015 was created out of the KNHIS-HSC database (*n* = 510,638), applying random number order to reduce probable selection bias. We removed probable participants who hold an RA diagnostic code and/or a prescription fill for a DMARD at any time during the study period (*n* = 78,040). 

To create the minimized differences between the RA and control groups in the baseline demographic and clinical features, propensity score matching was fulfilled for age, sex, income, and residential region, and patients with RA were individually matched with control participants based on close propensity score points. The index date of the control group followed the index date of their matched patient with RA. Through the matching steps, 420,318 control members were eventually unmatched and eliminated. Therefore, 3070 patients with RA were matched (1:4) with 12,280 controls. Then, we evaluated the cancer incidence (newly appointed ICD-10 codes for the designated cancers) in the RA and comparison groups between every participant’s index date and 31 December 2015. 

### 2.6. Covariates

All people were classified into 10 age groups (5-year intervals) and 5 income groups (class 1 [lowest income] to class 5 [highest income]) [15]. The residential regions were categorized into 16 on the basis of administrative district and were regrouped into urban and rural areas [15]. Obesity status was determined following the body mass index (BMI [kg/m^2^]; <18.5, ≥18.5 to <23, ≥23 to <25, ≥25 to <30, and ≥30 as underweight, normal weight, overweight, obese I, and obese II, respectively); alcohol consumption (<1 time or ≥1 time per week); and smoking status (non-, past-, and current- smokers) were graded similar to a previous study [15].

The total cholesterol (mg/dL), fasting blood glucose (mg/dL), and blood pressure (DBP and SBP, both in mmHg) were estimated. The Charlson Comorbidity Index (CCI) was a sum score from 0–29 to measure disease burden based on 17 potential comorbidities but without including cancer and rheumatic disease [18]. As comorbid diseases can influence the association of RA with cancer incidence, data on comorbidities were gathered, and they were adjusted as covariables to make the minimized potential confounding effects.

### 2.7. Statistical Analysis

The propensity score was generated using multivariable logistic regression for the four baseline covariates (age, sex, income, and residential region). In the propensity score matching steps, a greedy, nearest-neighbor matching algorithm was utilized to constitute pairs of RA patients and controls [19]. Standardized mean difference was employed to examine the balance of covariate distribution between the two groups. To conduct bias reduction, the intergroup balance was ascertained from the absolute standardized differences of covariates from before to after matching, and an absolute standardized difference <0.20 indicated a good balance for a particular covariate [20]. Categorical data are described as numbers with percentages.

Stratified Cox proportional hazard models stratified by income, age, sex, and residential area were exploited to assess the hazard ratios (HRs) and 95% confidence intervals (CIs) for various incident cancers among the RA patients and then compared with those of the controls. The proportional hazard assumptions were demonstrated by building log-minus-log plots, and no violations of these assumptions were identified (Supplementary Figure S1). An unadjusted crude model, model 1 (adjusted for obesity, smoking, alcohol consumption, and CCI scores), and model 2 (adjustments from model one and further adjustments for fasting blood glucose, SBP, DBP, and total cholesterol) were analyzed. 

For the subgroup analyses, we categorized the people by age (<60 years and ≥60 years), sex (male and female). Age-based subgroups were established based on median values. The Kaplan–Meier analysis with log-rank test was performed to determine the cumulative possibility of incident cancers in the RA group in comparison with the control group.

Two-tailed analyses were performed, and significance was defined as a *p*-value less than 0.05. The SAS version 9.4 (SAS Institute Inc., Cary, NC, USA) was used for all statistical analyses.

## 3. Results

### 3.1. Baseline Characteristics

**Figure 1:**
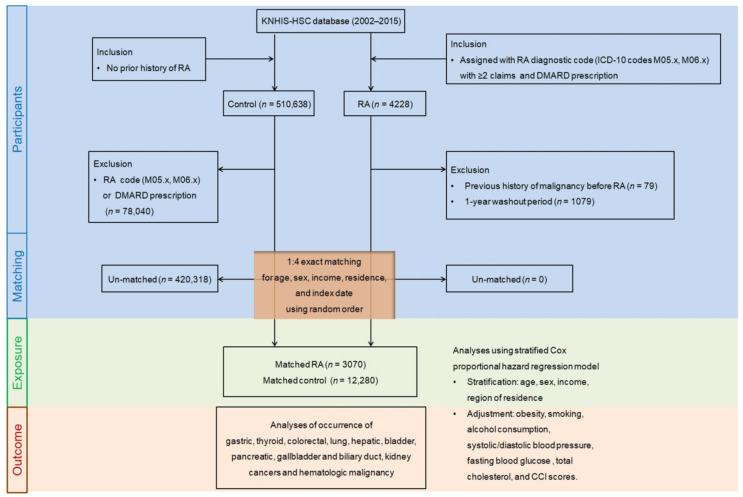
A schematic illustration of the participant selection process in this study. From a total sample of 514,866 patients, 3070 rheumatoid arthritis (RA) participants were matched with 12,280 controls based on age, sex, income, and residential region. Abbreviation: CCI, Charlson comorbidity index.

A total of 3070 participants with RA and 12,280 matched controls were finally enrolled in the analysis among 4228 newly diagnosed patients with RA between 2002 and 2015 (Figure 1). Males and females accounted for 26.4% and 73.6%, respectively, of the RA group, which showed non-smoker predominance (82.2%). The baseline characteristics of the RA and control groups subsequent to propensity score matching are summarized in Table 1, and the demographics of age, sex, economic level, and residential region are the same in both (standardized difference = 0). A balance of covariates was accomplished between those corresponding groups, except for the CCI score, since the RA group had a larger proportion of participants showing a CCI score of 1 or higher than in the comparison group (41.7% vs. 24.0%). Other features were equivalent between both RA and comparison groups (standardized difference ≤ 0.2).

### 3.2. Incidence of Malignancies in the RA Group and Control Group

Overall, malignancies occurred in 170 (5.5%) and 662 (5.4%) participants in the RA group (*n* = 3070) and without the RA group (*n* = 12,280), respectively. In the RA group, the five most common cancers were lung, thyroid, gastric, colorectal, and hepatic cancer (1.77, 1.64, 1.59, 1.26, and 0.65 incidence rate per 1000 person-years, respectively). In the control group, colorectal cancer was the most common (1.81), followed by thyroid (1.61) gastric (1.45), lung (1.03), and hepatic (0.66) cancer.

### 3.3. Association of the Incidence of Malignancy in the RA and Control Groups

The incidence rates of overall malignancy in the RA and control counterpart groups were 8.12 and 7.86 per 1000 person-years, respectively. After full adjustment, the HR of overall malignancy did not differ significantly between the RA and control groups (HR 1.02, 95% CI: 0.86–1.21; *p* = 0.819, Table 2). 

**Figure 2:**
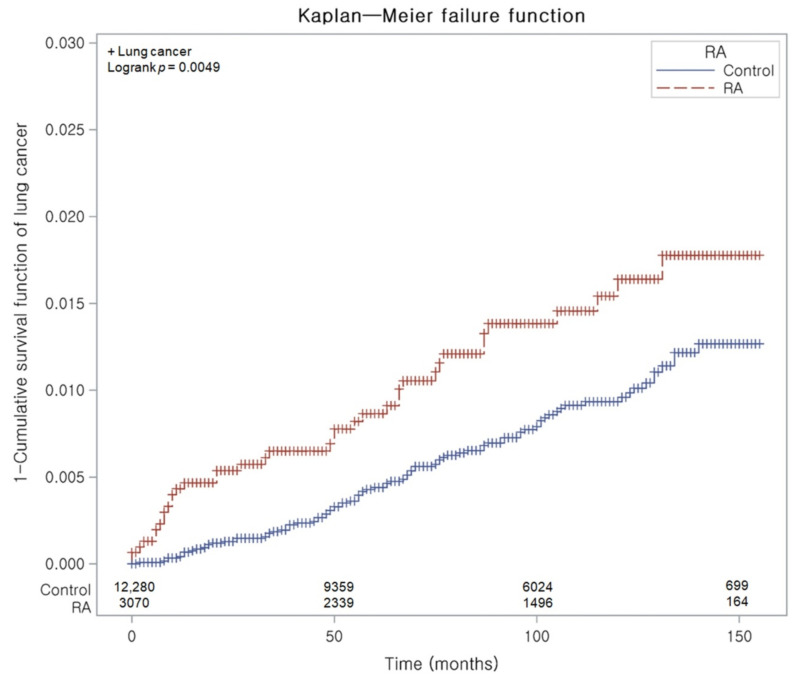
Kaplan–Meier probability of the incidence of lung cancer in the rheumatoid arthritis (RA) population and in controls during a 14-year period commencing from the index date.

There were 1.77 and 1.03 lung cancer events per 1000 person-year in the RA and non-RA cohorts, respectively (difference in incidence rates: HR 0.74, 95% CI: 0.22–1.25). After adjustment for all demographics and medical comorbidities, the Cox regression analysis disclosed that patients suffering RA showed a greater probability of lung cancer than the control counterparts (HR 1.63, 95% CI 1.11–2.40; *p* = 0.014). The Kaplan–Meier analysis with log-rank test displayed a more enhanced chance of lung cancer in patients suffering RA than in those without RA during a 14-year period commencing from the index date (*p* = 0.0049; Figure 2).

As age and sex are related with the development of both RA and lung cancer, the stratification of patients in respect to sex and age was additionally conducted to verify the relevance between RA and the development of lung cancer. In the sex-stratified subgroup analyses (Table 3), men in the RA group, compared with men in the comparison counterpart, had a greater likelihood of both thyroid and lung cancer (HR 5.15, 95% CI: 1.48–17.95, *p* = 0.010 and HR 1.86, 95% CI: 1.09–3.18, *p* = 0.022, respectively) but showed a lower likelihood of colorectal cancer (HR 0.37, 95% CI: 0.17–0.80, *p* = 0.012). However, there were no significant differences between the female participants in both the RA and comparison groups with regard to the likelihood of any malignancy.

In the age-stratified subgroup analyses (Table 4), patients in the RA group who were either younger than <60 or aged ≥60 years had a more prominent probability for lung cancer than those in the comparison counterpart in the crude model (HR 2.01, 95% CI: 1.01–4.02, *p* = 0.048 and HR 1.64, 95% CI: 1.04–2.60, *p* = 0.033, respectively). However, the analysis in the fully adjusted Model 2 revealed no significant associations (HR 2.00, 95% CI: 0.99–4.04, *p* = 0.053 and HR 1.50, 95% CI: 0.94–2.39, *p* = 0.089).

## 4. Discussion

In this nationwide cohort study, we did not find any remarkable differences in the overall cancer incidence between participants with or without RA (0.81% and 0.79% [8.12 and 7.86 per 1000 person-years]). This is consistent with previous studies from Korea (0.27–0.97% [2.7–9.7% per 1000 person-years]) [12,13,14], Japan (0.8%) [4], and worldwide (3.8–9.3 per 1000 person-years) among RA patients [6,7]. Our findings match those of previous studies [6,9], which have shown that the overall incidence of malignancy does not seem to be meaningfully intensified among patients with RA. The few studies with contrasting outcomes reported single-hospital-based outcomes [21,22], and the discrepant findings could be attributed to their insufficient cohort size and possible selection bias arising from the different patient characteristics depending on the primary, secondary, or tertiary hospital territory. Although a small raise in the general risk of cancer in patients suffering RA was detected in meta-analyses (pooled standardized incidence ratio 1.09, 95% CI: 1.06–1.13), the risk is less likely to be equal across all sites, and there seems to be either a higher or lower risk trend for some organ-specific malignancies than the trends in the general population [6,9]. 

Indeed, we noted specific associations between RA and the likelihood for some organ-specific malignancies: patients with RA displayed a 1.63-fold greater likelihood (95% CI 1.11–2.40) of developing lung cancer than those without RA. This superior chance of lung cancer in patients suffering RA than in those without RA was demonstrated throughout a 14-year period commencing from the index date in this study. This result matches those of a meta-analysis on incident organ-specific cancers that found a more prominent risk for both lung cancer and lymphoma but a more decreased risk for colorectal and breast cancers in patients suffering RA in comparison with the general population [6].

Compared with female patients suffering RA, male patients with RA demonstrate a higher incidence of cancer, which indicates a sex-specific difference in the RA-associated cancer risk [21]. In the present study, in comparison with female patients suffering RA, male patients with RA exhibited a 2.09- and 4.90-fold higher odds (95% CI: 1.25–3.50 and 1.49–16.07) for lung and thyroid cancer, respectively. Conversely, no similar association was found in female patients with RA or in the age-stratified subgroups. Our results are in part corresponding to those of a preceding meta-analysis, which described an elevated risk for lung cancer in male patients with RA [21]. However, there is limited information on association between male patients with RA and high likelihood of thyroid cancers, since this has not been a universal finding in other studies, usually from Western countries [21]. Indeed, South Korea has the highest incidence rate of thyroid cancer worldwide, with 63.4 per 100,000 in 2012 [23]. The finding of thyroid cancer as one of the top-ranking cancers in Korean RA patients has been previously reported [12]; the authors considered this observation might be the high number of screening examinations for thyroid cancers, which may lead to a high detection rate [23]. Some studies indicate that thyroid disease frequently involves Asian patients with RA and may increase the 2.5-fold more risk of malignancy in those people [12,13,24], while suggesting underlying thyroid disease may herald the risk of malignancy [13,24]. 

In the present study, compared with the male participants without RA, male patients suffering RA exhibited a 63% lower likelihood (95% CI 0.17–0.80) for colorectal cancer. A prospective study in Korea also found that patients suffering RA carried a lower risk of colorectal cancers in comparison with the general population [4]. This consistent finding appears to be inspiring in Korean RA patients, because colorectal cancer has been the second highest malignancy and major cause of death in South Korea [25]. The reduced risk might be involved in frequent medication of non-steroidal anti-inflammatory drugs (NSAIDs), and previously aspirin, in patients suffering RA. NSAIDs have been used to deal with symptomatic pain and for inflammation control in patients suffering RA. Based on their use in the general population, it is well known that NSAID use is associated with a reduced risk of colorectal cancer [26], and it may be feasible that this effect extends to patients with RA [27]. As we did not investigate the medication of NSAIDs in the study, we could not confirm the pharmacotherapeutic effect in male patients suffering RA. Nonetheless, the relatively lower incidence of specific malignancies, especially colorectal cancer, in male patients suffering RA than in the general male population may be of great clinical importance that could confer psychological relief. 

Furthermore, it is important to investigate the difference in the site-specific cancer risk in patients with RA in comparison to that in the general population. One theory is that certain cancers and RA might share risk factors, which may partially account for the elevated likelihood for lung cancer that was observed in the present study. The causal relation between tobacco use and lung cancer has been well corroborated, and it is evaluated that smoking is accountable for up to 85% of the cases of lung cancer [28,29]. Moreover, it is widely acknowledged that smoking also constitutes a risk factor for RA, which promotes the risk of RA by approximately 40% [29,30]. The elevated risk of RA relevant to the genetic risk factors for immune-mediated diseases (the shared epitope of HLA-DR) is more affected by the environmental factor (smoking) in the susceptible individuals than never smokers with the shared epitope genes [10]. Therefore, smoking may be a shared risk factor for both lung cancer and RA in a subset of the population [10,29]. However, the theory of shared risk factors might not explain the increased risk for all cancers in patients with RA, because one current study indicates a significant link between RA and lung cancer despite never-smokers making up the majority (84.5%) of participants [13]. Moreover, chronic inflammatory/immune stimulation or impaired immune surveillance has been suggested to facilitate the risk of specific tumorigenesis (e.g., lymphoma) in patients with RA [31]. 

This current study has some limitations that need to be stated. First, as we enrolled participants on the basis of diagnostic codes and contained only Korean subjects, the influence of unmeasured confounders might not be excluded completely. The prescription of DMARDs took account for more than 99%, whereas the biologic agents were rarely seen because the biologic agents were not covered by the Korean National Health Insurance Service during the study period, consistent with the previous studies [16,17], which might be confounders. Second, information regarding certain risk factors for malignancy, such as family history, dietary factors, and hormone therapy, or cancer-related genomic data were unavailable in the KNHIS-HSC dataset; however, the possibility of missing data was not taken into account. It may be challenging to explore the risk of malignancy in patients with RA due to the requirement for long-term follow-up in cancer data; accordingly, the cancer risk may not be the same for all patients with RA due to demographic, geographic, and national differences as well as the diverse treatment regimens and cancer outcomes. Third, in this study, skin cancer was not included in the analysis because skin cancer had not been reported in the cancer lists among RA patients. Similarly, one prospective study from Korea also did not comment on skin cancer during the follow-up [13]. 

Nonetheless, one of the chief strengths of the present study is that this research is based on a large, representative, nationwide population database covering proper identification of case and control patients, which makes the study results more generalizable. As the KNHIS-HSC data include information from all the clinics and hospitals within South Korea without any exception, no details of the medical history would have been missed during the follow-up duration. We widely considered possible confounders, including age and sex, among patients with RA with regard to the incidence of malignancies. Full adjustment of socioeconomic status and possible lifestyle-based risk factors and comorbidities related to malignancies or RA (e.g., total cholesterol, alcohol, smoking, blood pressure, obesity, and fasting blood glucose) are supplementary strengths of this study.

## 5. Conclusions

Our data provide information on likelihood trends for various incident cancers to make patients aware of potential malignancies, which may motivate the alert to their personal health care and encourage prevention in the patients with RA. Korean patients suffering RA may have a higher likelihood of lung cancer than those without RA all throughout the follow-up periods; Korean male patients with RA seem susceptible to lung and thyroid cancers but are at lower risk ofcolorectal cancers. Therefore, patients with RA need to be monitored carefully to ensure early detection and administration of appropriate cancer treatment. 

## Figures and Tables

**Table 1:** General characteristics of participants.

Characteristics	Total Participants		
	Rheumatoid Arthritis (*n*, %)	Control (*n*, %)	SMD
Total number	3070 (100.0)	12,280 (100.0)	
Age (years old)			0.00
40–44	109 (3.6)	436 (3.6)	
45–49	363 (11.8)	1452 (11.8)	
50–54	662 (21.6)	2648 (21.6)	
55–59	584 (19.0)	2336 (19.0)	
60–64	539 (17.6)	2156 (17.6)	
65–69	414 (13.5)	1656 (13.5)	
70–74	235 (7.7)	940 (7.7)	
75–79	127 (4.1)	508 (4.1)	
80–84	32 (1.0)	128 (1.0)	
85+	5 (0.2)	20 (0.2)	
Sex			0.00
Male	810 (26.4)	3240 (26.4)	
Female	2260 (73.6)	9040 (73.6)	
Income			0.00
1 (lowest)	514 (16.7)	2056 (16.7)	
2	463 (15.1)	1852 (15.1)	
3	533 (17.4)	2132 (17.4)	
4	646 (21.0)	2584 (21.0)	
5 (highest)	914 (29.8)	3656 (29.8)	
Region of residence			0.00
Urban	1320 (43.0)	5280 (43.0)	
Rural	1750 (57.0)	7000 (57.0)	
Obesity ^†^			0.06
Underweight	59 (1.9)	287 (2.3)	
Normal	1169 (38.1)	4394 (35.8)	
Overweight	802 (26.1)	3329 (27.1)	
Obese I	952 (31.0)	3877 (31.6)	
Obese II	88 (2.9)	393 (3.2)	
Smoking status			0.04
Nonsmoker	2524 (82.2)	10,254 (83.5)	
Past smoker	209 (6.8)	749 (6.1)	
Current smoker	337 (11.0)	1277 (10.4)	
Alcohol consumption			0.05
<1 time a week	2476 (80.7)	9676 (78.8)	
≥1 time a week	594 (19.4)	2604 (21.2)	
Systolic blood pressure			0.06
<120 mmHg	1009 (32.9)	3982 (32.4)	
120–139 mmHg	1472 (48.0)	5642 (45.9)	
≥140 mmHg	589 (19.2)	2656 (21.6)	
Diastolic blood pressure			0.05
<80 mmHg	1511 (49.2)	5742 (46.8)	
80–89 mmHg	1049 (34.2)	4304 (35.1)	
≥90 mmHg	510 (16.6)	2234 (18.2)	
Fasting blood glucose			0.08
<100 mg/dL	2138 (69.6)	8126 (66.2)	
100–125 mg/dL	724 (23.6)	3192 (26.0)	
≥126 mg/dL	208 (6.8)	962 (7.8)	
Total cholesterol			0.03
<200 mg/dL	1595 (52.0)	6254 (50.9)	
200–239 mg/dL	1033 (33.7)	4146 (33.8)	
≥240 mg/dL	442 (14.4)	1880 (15.3)	
CCI score ^‡^			0.38
0	1791 (58.3)	9327 (76.0)	
1	801 (26.1)	1832 (14.9)	
≥2	478 (15.6)	1121 (9.1)	
All of cancers	170 (5.5)	662 (5.4)	0.01
Gastric cancer	34 (1.1)	124 (1.0)	0.01
Thyroid cancer	35 (1.1)	138 (1.1)	0.00
Colorectal cancer	27 (0.9)	155 (1.3)	0.04
Lung cancer	38 (1.2)	89 (0.7)	0.05
Hepatic cancer	14 (0.5)	57 (0.5)	0.00
Bladder cancer	7 (0.2)	23 (0.2)	0.01
Pancreatic cancer	7 (0.2)	24 (0.2)	0.01
Gallbladder/biliary duct	8 (0.3)	41 (0.3)	0.01
Kidney cancer	3 (0.1)	18 (0.2)	0.01
Hematologic malignancy	10 (0.3)	43 (0.4)	0.00

Abbreviation: SMD, Standardized mean difference; CCI, Charlson comorbidity index. Significance at *p* < 0.05. ^†^ Obesity (BMI, body mass index, kg/m^2^) was categorized as <18.5 (underweight), ≥18.5 to <23 (normal), ≥23 to <25 (overweight), ≥25 to <30 (obese I), and ≥30 (obese II). ^‡^ CCI scores were calculated without cancer, metastatic cancer, and rheumatic disease.

**Table 2:** Crude and adjusted hazard ratios of RA for various cancers.

Dependent Variable	IR/1000 PY		IRD/1000 PY(95% CI)	Hazard Ratios for Cancers (95% CI)					
	RA (*n* = 3070)	Control (*n* = 12,280)		Crude ^†^	*p*	Model 1 ^†,‡^	*p*	Model 2 ^†,§^	*p*
All cancers (*n* = 832)	8.12	7.86	0.26 (−1.09–1.61)	1.04 (0.88–1.23)	0.637	1.02 (0.86–1.21)	0.825	1.02 (0.86–1.21)	0.819
Gastric (*n* = 158)	1.59	1.45	0.14 (−0.43–0.72)	1.11 (0.76–1.62)	0.594	1.11 (0.76–1.63)	0.600	1.12 (0.76–1.64)	0.565
Thyroid (*n* = 173)	1.64	1.61	0.03 (−0.57–0.63)	1.01 (0.70–1.47)	0.951	1.01 (0.70–1.47)	0.949	1.01 (0.69–1.46)	0.980
Colorectal (*n* = 182)	1.26	1.81	−0.55 (−1.16–0.07)	0.69 (0.46–1.04)	0.072	0.68 (0.45–1.03)	0.070	0.68 (0.45–1.02)	0.062
Lung (*n* = 127)	1.77	1.03	0.74 (0.22–1.25)	1.74 (1.19–2.55)	0.004 *	1.64 (1.11–2.41)	0.013 *	1.63 (1.11–2.40)	0.014 *
Hepatic (*n* = 71)	0.65	0.66	−0.01 (−0.39–0.37)	1.01 (0.56–1.82)	0.971	0.95 (0.52–1.73)	0.875	0.96 (0.53–1.76)	0.903
Bladder (*n* = 30)	0.33	0.27	0.06 (−0.19–0.31)	1.25 (0.54–2.91)	0.610	1.24 (0.53–2.91)	0.616	1.27 (0.54–2.97)	0.585
Pancreatic (*n* = 31)	0.32	0.28	0.05 (−0.21–0.30)	1.16 (0.50–2.70)	0.724	1.21 (0.52–2.83)	0.663	1.23 (0.52–2.89)	0.638
GB/BD (*n* = 49)	0.37	0.47	−0.10 (−0.42–0.21)	0.77 (0.36–1.65)	0.506	0.71 (0.33–1.54)	0.390	0.70 (0.32–1.51)	0.361
Kidney (*n* = 21)	0.14	0.21	−0.07 (−0.28–0.14)	0.66 (0.20–2.25)	0.508	0.67 (0.20–2.30)	0.523	0.68 (0.20–2.35)	0.543
Hematologic (*n* = 53)	0.46	0.50	−0.03 (−0.36–0.30)	0.94 (0.47–1.87)	0.861	0.87 (0.43–1.75)	0.694	0.87 (0.43–1.76)	0.705

Abbreviation: RA, rheumatoid arthritis; IR, incidence rate; PY, person-year; IRD, incidence rate difference; CI, confidence interval; GB, gallbladder; BD, biliary duct. * Stratified Cox proportional hazard regression model, Significance at *p* < 0.05. ^†^ Models were stratified by age, sex, income, and region of residence. ^‡^ Model 1 was adjusted for obesity, smoking, alcohol consumption, and Charlson comorbidity index (CCI) scores. ^§^ Model 2 was adjusted for model 1 with systolic blood pressure, diastolic blood pressure, fasting blood glucose, and total cholesterol.

**Table 3:** Subgroup analysis of crude and adjusted hazard ratios of RA for various cancers by sex.

Dependent Variable	IR/1000 PY		IRD/1000 PY (95% CI)	Hazard Ratios for Cancers (95% CI)					
	RA	Control		Crude ^†^	*p*	Model 1 ^†,‡^	*p*	Model 2 ^†,§^	*p*
Men (*n* = RA: 810, Control: 3240)									
All cancers (*n* = 309)	14.11	12.20	1.91 (−1.62–5.44)	1.17 (0.90–1.54)	0.248	1.12 (0.85–1.47)	0.422	1.13 (0.86–1.49)	0.376
Gastric (*n* = 70)	3.44	2.63	0.81 (−0.84–2.45)	1.35 (0.78–2.34)	0.285	1.32 (0.75–2.32)	0.330	1.35 (0.77–2.37)	0.299
Thyroid (*n* = 11)	1.20	0.25	0.96 (0.31–1.60)	4.90 (1.49–16.07)	0.009 *	5.19 (1.53–17.64)	0.008 *	5.15 (1.48–17.95)	0.010 *
Colorectal (*n* = 78)	1.40	3.52	−2.12 (−3.85–−0.40)	0.38 (0.18–0.83)	0.015 *	0.38 (0.17–0.82)	0.014 *	0.37 (0.17–0.80)	0.012 *
Lung (*n* = 65)	4.41	2.11	2.29 (0.73–3.86)	2.09 (1.25–3.50)	0.005 *	1.83 (1.07–3.11)	0.026 *	1.86 (1.09–3.18)	0.022 *
Hepatic (*n* = 39)	1.00	1.67	−0.67 (−1.88–0.54)	0.62 (0.24–1.58)	0.317	0.60 (0.23–1.62)	0.314	0.63 (0.24–1.71)	0.367
Bladder (*n* = 19)	0.60	0.79	−0.19 (−1.03–0.66)	0.78 (0.23–2.69)	0.698	0.77 (0.22–2.66)	0.674	0.79 (0.22–2.75)	0.705
Pancreatic (*n* = 13)	0.60	0.49	0.11 (−0.59–0.80)	1.20 (0.33–4.39)	0.778	1.22 (0.31–4.91)	0.775	1.11 (0.25–4.95)	0.893
GB/biliary duct (*n* = 15)	0.80	0.54	0.26 (−0.49–1.01)	1.44 (0.45–4.54)	0.538	1.68 (0.49–5.76)	0.407	1.56 (0.44–5.53)	0.495
Kidney (*n* = 10)	0.40	0.39	0.01 (−0.61–0.62)	1.03 (0.22–4.85)	0.971	0.99 (0.20–4.79)	0.987	1.02 (0.20–5.09)	0.982
Hematologic (*n* = 18)	0.80	0.69	0.11 (−0.71–0.93)	1.19 (0.39–3.63)	0.758	0.90 (0.29–2.84)	0.862	1.03 (0.32–3.35)	0.956
Women (*n* = RA: 2260, Control: 9040)									
All cancers (*n* = 523)	6.33	6.53	−0.20 (−1.59–1.19)	0.97 (0.78–1.20)	0.773	0.95 (0.77–1.18)	0.659	0.95 (0.76–1.18)	0.618
Gastric (*n* = 88)	1.03	1.08	−0.05 (−0.61–0.51)	0.94 (0.55–1.60)	0.819	0.95 (0.56–1.62)	0.852	0.95 (0.56–1.62)	0.846
Thyroid (*n* = 162)	1.77	2.03	−0.27 (−1.03–0.50)	0.87 (0.58–1.30)	0.491	0.86 (0.58–1.29)	0.472	0.86 (0.57–1.28)	0.457
Colorectal (*n* = 104)	1.22	1.28	−0.06 (−0.67–0.55)	0.95 (0.58–1.55)	0.831	0.94 (0.58–1.54)	0.811	0.93 (0.57–1.51)	0.756
Lung (*n* = 62)	0.97	0.70	0.27 (−0.20–0.74)	1.42 (0.80–2.52)	0.229	1.48 (0.83–2.63)	0.181	1.51 (0.85–2.69)	0.159
Hepatic (*n* = 32)	0.54	0.35	0.20 (−0.14–0.53)	1.57 (0.72–3.39)	0.254	1.45 (0.66–3.16)	0.357	1.43 (0.64–3.16)	0.383
Bladder (*n* = 11)	0.24	0.11	0.14 (−0.06–0.33)	2.26 (0.66–7.72)	0.194	2.28 (0.64–8.19)	0.205	2.43 (0.66–8.94)	0.182
Pancreatic (*n* = 18)	0.24	0.21	0.03 (−0.22–0.28)	1.14 (0.37–3.45)	0.822	1.11 (0.37–3.39)	0.85	1.14 (0.37–3.52)	0.814
GB/biliary duct (*n* = 34)	0.24	0.45	−0.21 (−0.56–0.13)	0.53 (0.19–1.51)	0.234	0.51 (0.17–1.49)	0.217	0.50 (0.17–1.46)	0.206
Kidney (*n* = 11)	0.06	0.15	−0.09 (−0.29–0.11)	0.38 (0.05–3.00)	0.362	0.41 (0.05–3.23)	0.394	0.40 (0.05–3.22)	0.387
Hematologic (*n* = 35)	0.36	0.44	−0.08 (−0.43–0.27)	0.82 (0.34–1.99)	0.666	0.82 (0.34–1.99)	0.661	0.79 (0.32–1.93)	0.601

Abbreviation; RA, rheumatoid arthritis; IR, incidence rate; PY, person-year; IRD, incidence rate difference; CI, confidence interval; GB, gallbladder. * Stratified Cox proportional hazard regression model, Significance at *p* < 0.05. ^†^ Models were stratified by age, sex, income, and region of residence. ^‡^ Model 1 was adjusted for obesity, smoking, alcohol consumption, and Charlson comorbidity index (CCI) scores. ^§^ Model 2 was adjusted for model 1 with systolic blood pressure, diastolic blood pressure, fasting blood glucose, and total cholesterol.

**Table 4:** Subgroup analysis of Crude and adjusted hazard ratios of RA for various cancers by age.

Dependent Variable	IR/1000 PY		IRD/1000 PY(95% CI)	Hazard Ratios for Cancers (95% CI)					
	RA	Control		Crude ^†^	*p*	Model 1 ^†,‡^	*p*	Model 2 ^†,§^	*p*
Age < 60 (*n* = RA: 1718, Control: 6872)									
All cancers (*n* = 367)	4.93	5.74	−0.81 (−2.24–0.62)	0.86 (0.66–1.13)	0.281	0.83 (0.64–1.09)	0.182	0.83 (0.63–1.08)	0.171
Gastric (*n* = 53)	0.60	0.84	−0.24 (−0.78–0.29)	0.71 (0.33–1.50)	0.369	0.65 (0.31–1.39)	0.266	0.66 (0.31–1.40)	0.278
Thyroid (*n* = 127)	1.65	1.97	−0.32 (−1.15–0.51)	0.83 (0.53–1.32)	0.435	0.83 (0.52–1.31)	0.423	0.82 (0.52–1.30)	0.401
Colorectal (*n* = 77)	0.74	1.25	−0.51 (−1.15–0.13)	0.60 (0.31–1.16)	0.127	0.59 (0.31–1.16)	0.126	0.60 (0.31–1.16)	0.127
Lung (*n* = 36)	0.89	0.45	0.45 (0.01–0.88)	2.01 (1.01–4.02)	0.048 *	1.99 (0.99–4.00)	0.054	2.00 (0.99–4.04)	0.053
Hepatic (*n* = 29)	0.52	0.41	0.11 (−0.28–0.50)	1.29 (0.55–3.03)	0.554	1.16 (0.48–2.80)	0.748	1.21 (0.50–2.93)	0.671
Bladder (*n* = 11)	0.15	0.17	−0.02 (−0.26–0.22)	0.90 (0.20–4.18)	0.896	0.58 (0.12–2.92)	0.508	0.60 (0.11–3.26)	0.551
Pancreatic (*n* = 12)	0.30	0.15	0.15 (−0.10–0.40)	1.99 (0.60–6.62)	0.260	2.22 (0.64–7.71)	0.210	2.20 (0.62–7.74)	0.221
GB/biliary duct (*n* = 13)	0.15	0.20	−0.06 (−0.32–0.21)	0.73 (0.16–3.28)	0.679	0.79 (0.17–3.76)	0.765	0.65 (0.13–3.18)	0.594
Kidney (*n* = 11)	0.15	0.17	−0.02 (−0.26–0.22)	0.87 (0.19–4.02)	0.858	1.00 (0.21–4.72)	0.999	0.89 (0.19–4.25)	0.882
Hematologic (*n* = 21)	0.07	0.37	−0.30 (−0.63–0.04)	0.20 (0.03–1.51)	0.119	0.17 (0.02–1.29)	0.086	0.15 (0.02–1.17)	0.070
Age ≥ 60 (*n* = RA: 1352, Control: 5408)									
All cancers (*n* = 465)	13.53	11.38	2.16 (−0.54–4.85)	1.19 (0.96–1.49)	0.112	1.17 (0.94–1.46)	0.161	1.18 (0.94–1.46)	0.150
Gastric (*n* = 105)	3.26	2.45	0.81 (−0.44–2.06)	1.34 (0.86–2.09)	0.194	1.40 (0.89–2.19)	0.145	1.41 (0.90–2.21)	0.136
Thyroid (*n* = 46)	1.62	1.02	0.60 (−0.22–1.42)	1.59 (0.84–3.02)	0.158	1.56 (0.82–2.97)	0.177	1.56 (0.82–2.97)	0.179
Colorectal (*n* = 105)	2.12	2.72	−0.60 (−1.85–0.64)	0.75 (0.45–1.27)	0.290	0.75 (0.45–1.27)	0.289	0.73 (0.43–1.24)	0.244
Lung (*n* = 91)	3.23	2.00	1.23 (0.07–2.38)	1.64 (1.04–2.60)	0.033*	1.51 (0.95–2.40)	0.084	1.50 (0.94–2.39)	0.089
Hepatic (*n* = 42)	0.87	1.07	−0.21 (−0.99–0.57)	0.83 (0.37–1.87)	0.653	0.78 (0.34–1.79)	0.559	0.81 (0.35–1.88)	0.631
Bladder (*n* = 19)	0.62	0.43	0.19 (−0.34–0.72)	1.47 (0.53–4.09)	0.460	1.44 (0.51–4.01)	0.490	1.46 (0.52–4.10)	0.469
Pancreatic (*n* = 19)	0.37	0.49	−0.12 (−0.65–0.41)	0.75 (0.22–2.57)	0.645	0.70 (0.20–2.45)	0.572	0.64 (0.17–2.37)	0.500
GB/biliary duct (*n* = 36)	0.74	0.92	−0.18 (−0.90–0.54)	0.79 (0.33–1.90)	0.598	0.75 (0.31–1.81)	0.516	0.73 (0.30–1.80)	0.499
Kidney (*n* = 10)	0.12	0.28	−0.15 (−0.53–0.23)	0.45 (0.06–3.54)	0.446	0.54 (0.06–4.53)	0.568	0.50 (0.06–4.67)	0.547
Hematologic (*n* = 32)	1.11	0.71	0.41 (−0.28–1.09)	1.58 (0.73–3.43)	0.244	1.52 (0.69–3.32)	0.298	1.52 (0.69–3.35)	0.299

Abbreviation; RA, rheumatoid arthritis; IR, incidence rate; PY, person-year; IRD, incidence rate difference; CI, confidence interval; GB, gallbladder. * Stratified Cox proportional hazard regression model, Significance at *p* < 0.05. ^†^ Models were stratified by age, sex, income, and region of residence. ^‡^ Model 1 was adjusted for obesity, smoking, alcohol consumption, and Charlson comorbidity index (CCI) scores. ^§^ Model 2 was adjusted for model 1 with systolic blood pressure, diastolic blood pressure, fasting blood glucose, and total cholesterol.

## Data Availability

All data are available from the database of the National Health Insurance Sharing Service (NHISS) https://nhiss.nhis.or.kr/ (accessed on 23 August 2021) NHISS allows access to all of this data for any researcher who promises to follow the research ethics at some cost. Those seeking access to this articles’ data can download it from the website after promising to follow the research ethics.

## Supplementary Materials

The following supporting information can be downloaded at: https://www.mdpi.com/article/10.3390/jpm12060965/s1. Figure S1: The proportional hazard assumptions are demonstrated by building log-minus-log plots, and no violations of these assumptions are identified.
