# Supplementary material for: Potential Cancer Risk in Patients with Rheumatoid Arthritis: A Longitudinal Korean Population-Based Analysis
Source: J Pers Med. 2022 Jun 13;12(6):965. doi: 10.3390/jpm12060965 (PMC9224951; doi:10.3390/jpm12060965)
Supplement: Supplementary file 1 [file jpm-12-00965-s001.zip › jpm-1746885-supplementary.pdf]

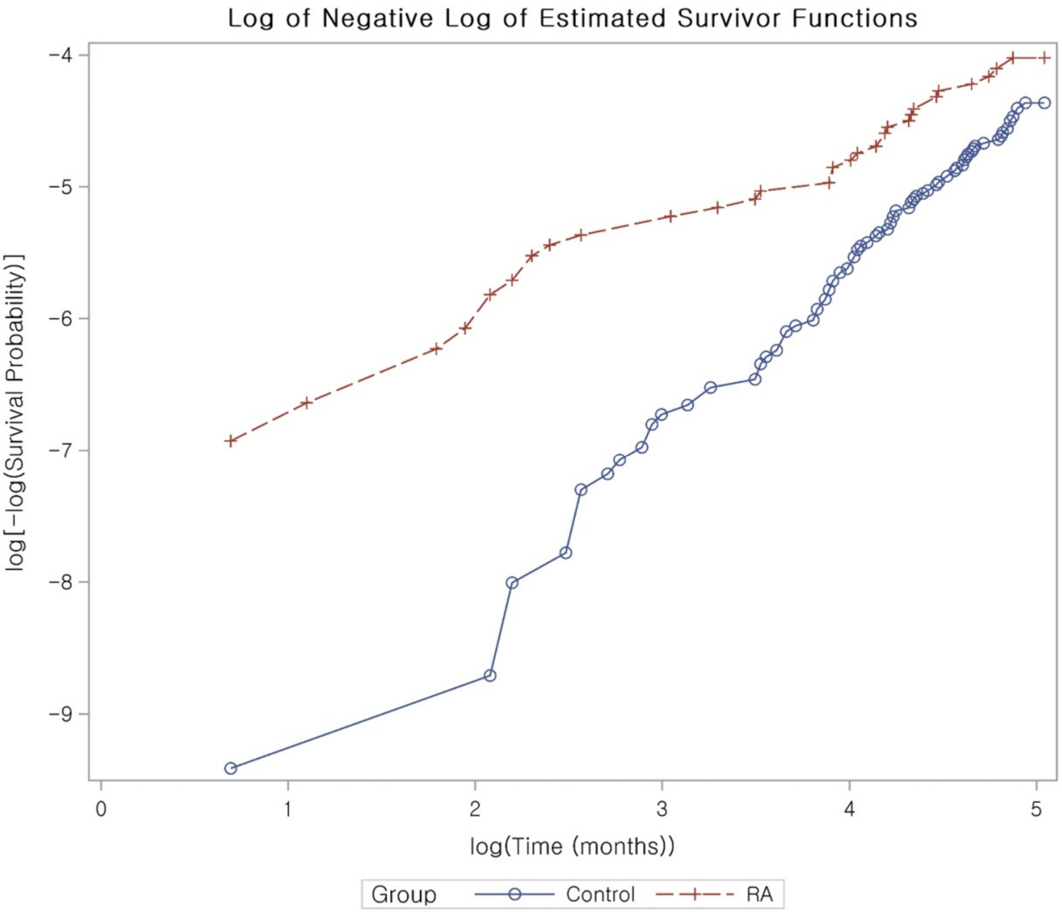

Supplementary Figure S1. The proportional hazard assumptions are demonstrated by building log-minus-log plots, and no violations of these assumptions are identified.
